# Innovative applications and future perspectives of chromatography-mass spectrometry in drug research

**DOI:** 10.3389/fphar.2025.1529468

**Published:** 2025-03-26

**Authors:** Hong Cai, Xue Xing, Ying Su, Chunhui Yang

**Affiliations:** Department of Clinical Laboratory, The Second Hospital of Dalian Medical University, Dalian, Liaoning, China

**Keywords:** chromatography-mass spectrometry, drug research, pharmacokinetics, drug metabolism, personalized medicine

## Abstract

Chromatography coupled with mass spectrometry (MS) has emerged as a cornerstone analytical technique in drug research. Over the years, advancements in chromatography-MS have significantly enhanced its capabilities, leading to improved sensitivity, specificity, and throughput. This review explores the innovative applications of chromatography-MS in drug research, particularly focusing on its role in drug absorption, distribution, metabolism, excretion (ADME), toxicity evaluation, and personalized medicine. It also addresses the future perspectives of this powerful technique, including challenges and potential solutions, and highlights how emerging trends such as high spatial resolution imaging and multimodal integration could revolutionize drug discovery and development. Through these innovations, chromatography-MS promises to contribute substantially to the development of more effective, safer, and personalized therapeutic interventions.

## 1 Introduction

Chromatography-MS has established itself as one of the most versatile and powerful analytical techniques in drug research (Purves et al., 2022). The integration of high-resolution chromatography with sensitive mass spectrometry has transformed the landscape of pharmaceutical analysis, enabling researchers to gain unprecedented insights into drug molecules (Tsujimoto et al., 2019). In particular, chromatography-MS has become indispensable in understanding critical aspects of drug behavior, such as drug pharmacokinetics, pharmacodynamics, metabolism, distribution, and excretion (ADME), and toxicity evaluation (Liu, 2022; Singh et al., 2020). The ability to resolve complex biological mixtures, detect trace components, and provide detailed molecular information has expanded the boundaries of drug research, offering researchers a precise toolkit to explore and evaluate drug mechanisms in ways that were previously unattainable.

The growing complexity of drug research, combined with the increasing demand for precision medicine, underscores the need for sophisticated analytical techniques capable of addressing the challenges of drug discovery, development, and personalized treatment (Zhou and Zhong, 2022). For example, in the early stages of drug development, the need for accurate pharmacokinetic and pharmacodynamic profiling is paramount to predicting a drug’s behavior in the body. To optimize drug efficacy and minimize risks, researchers must track how drugs are absorbed, distributed, metabolized, eliminated, as well as assess their toxicity (Son et al., 2024). However, the human body is a highly dynamic and complex system, with drug molecules interacting with various tissues, organs, and metabolic pathways in ways that can be difficult to predict. In this context, chromatography-MS offers a powerful solution for achieving the sensitivity and precision needed to dissect these complex processes.

Moreover, the field of personalized medicine has become increasingly important, aiming to tailor drug therapies to individual patients based on their unique genetic makeup, metabolism, and drug response profiles (Wang D. et al., 2023; Ye et al., 2024). Chromatography-MS plays a crucial role in this context by providing essential data for biomarker discovery, identifying genetic or metabolic factors that influence drug metabolism, and enabling the development of targeted therapies that maximize efficacy while minimizing adverse effects. The ability to identify and quantify individual metabolites, for instance, can help predict which patients are most likely to benefit from a specific drug or therapy, marking a significant step toward the realization of precision medicine (Schwarz et al., 2025).

The importance of chromatography-MS in drug research cannot be overstated, as it supports a wide range of applications-from early-stage drug discovery to clinical development and personalized treatment strategies (Luo J. et al., 2023). As technologies in chromatography and mass spectrometry continue to improve, the potential for chromatography-MS to revolutionize drug research expands, promising more accurate, faster, and more efficient methods for developing safer and more effective therapeutic interventions.

Given its importance, this review aims to highlight the innovative applications of chromatography-MS in drug research, shedding light on its role across various stages of drug development. This includes drug absorption, distribution, metabolism, excretion, and toxicity assessment, as well as emerging areas like drug deconvolution and personalized medicine. Furthermore, the review will address the current challenges faced in the application of chromatography-MS, and provide future perspectives on how advancements in this field can help overcome these obstacles. By identifying key areas where chromatography-MS is likely to continue evolving, this review aims to underscore the technique’s necessity and its potential to play an even greater role in shaping the future of drug discovery and development.

## 2 Principles and development of chromatography-MS

Chromatography-MS is an integrated analytical technique that combines the separation capabilities of chromatography with the molecular identification power of mass spectrometry. The success of this approach lies in its ability to simultaneously separate complex mixtures, identify individual components with high sensitivity, and provide detailed structural information (Ross et al., 2019). This combination allows for the analysis of a wide variety of drug-related samples, from biological fluids to tissues, and provides valuable insights into the behavior and mechanisms of drugs at both the molecular and cellular levels (Ruan et al., 2020). Below, we discuss the fundamental principles of chromatography and mass spectrometry, as well as the evolution of both technologies, which have contributed to the growing significance of chromatography-MS in drug research.

### 2.1 Chromatography: separation principle

Chromatography is a technique used to separate components of a mixture based on their different affinities for two phases: a stationary phase and a mobile phase (Broeckhoven and Desmet, 2022). The stationary phase is typically a solid or a liquid that is adhered to a solid support (e.g., silica or polymer beads), while the mobile phase is a liquid or gas that carries the sample through the stationary phase. As the sample moves through the column, components of the mixture interact differently with the stationary phase, causing them to move at different rates. This differential interaction results in the separation of the components in the sample, which can then be detected and analyzed (Wong, 2017). There are several types of chromatography commonly used in drug research:

(1) Liquid Chromatography (LC): LC, particularly high-performance liquid chromatography (HPLC) and ultra-high-performance liquid chromatography (UHPLC), is one of the most widely used techniques in drug research (Ma et al., 2022). LC is effective for separating a wide range of polar and non-polar compounds, including small molecules, peptides, and proteins. In HPLC, the mobile phase is a liquid, and the stationary phase is typically a solid or porous material packed into a column. UHPLC improves upon HPLC by using smaller particle sizes and higher pressure, allowing for faster separation and greater resolution. Additionally, UHPLC demonstrates superior capabilities in the separation of nonpolar lipid molecules, which is particularly advantageous given the challenges associated with analyzing such compounds using traditional chromatographic techniques. Lipids, particularly those with low polarity, often exhibit significant retention in conventional HPLC systems, leading to poor resolution and inefficient separation. However, UHPLC, with its advanced column technology and optimized mobile phase compositions, enables more precise lipid fractionation, thereby enhancing the accuracy of lipidomic studies. This advantage is crucial in drug research, where lipid metabolism plays a significant role in drug absorption, distribution, and cellular interactions (Holčapek et al., 2015). The ability of UHPLC to resolve complex lipid mixtures with high sensitivity makes it a preferred technique for studying lipid-based drug formulations, lipophilic drug metabolism, and biomarker discovery in lipidomics (Nambiar et al., 2021).
(2) Gas Chromatography (GC): GC is used primarily for volatile compounds. It involves the separation of a sample by passing it through a column with a stationary phase, with a gas (usually helium or nitrogen) as the mobile phase. GC is widely used in the analysis of small drug molecules, particularly those that are thermally stable and volatile (Tranchida, 2018).
(3) Two-Dimensional Chromatography (2D-LC): Two-dimensional chromatography combines two different chromatographic techniques to achieve even greater separation power. The first dimension often involves an LC method, while the second dimension may use a different technique, such as ion-exchange chromatography or reverse-phase chromatography. 2D-LC can resolve complex mixtures that would otherwise be difficult to separate with a single chromatographic step (He J. et al., 2020).
(4) Thin-Layer Chromatography (TLC): TLC is an older but still widely used technique for the rapid separation of small amounts of drug compounds. It involves the application of a sample onto a thin layer of stationary phase, typically silica gel, followed by the movement of a solvent up the plate, causing the separation of the components based on their affinity for the stationary phase (Shakibai et al., 2019).

In chromatography-MS, the separation achieved by chromatography is followed by ionization of the components and their detection by mass spectrometry. This separation ensures that the mass spectrometer is able to detect individual compounds in complex mixtures, even in very small concentrations, which is essential for studying drug molecules and their metabolites in biological samples.

### 2.2 MS: detection and quantification

MS is a technique used to identify the chemical structure and quantify the amount of a compound based on its mass-to-charge ratio (m/z). The process of mass spectrometry typically involves three main stages: ionization, mass analysis, and detection (Largy et al., 2022).

(1) Ionization: The first step in MS is the conversion of neutral molecules into charged particles, or ions, which can then be manipulated in an electric or magnetic field. Ionization techniques vary depending on the type of sample being analyzed. Common ionization methods include: 1) electrospray Ionization (ESI): ESI is widely used for the analysis of polar and ionic compounds, such as drugs and their metabolites. It generates charged droplets in a high electric field, from which ions are released as the solvent evaporates (Khristenko et al., 2023). 2) Atmospheric Pressure Chemical Ionization (APCI): APCI is used for less polar compounds and works by creating ions through chemical reactions between the sample and a reagent gas in a corona discharge (Kangasluoma et al., 2022). 3) Matrix-Assisted Laser Desorption/Ionization (MALDI): MALDI is typically used for the analysis of large biomolecules such as proteins and peptides. A laser is used to ionize the sample embedded in a matrix, producing ions that can be analyzed in the mass spectrometer (Engel et al., 2022).
(2) Mass Analysis: Once the sample is ionized, the ions are directed into the mass analyzer, where their mass-to-charge ratio (m/z) is measured. Several types of mass analyzers are used, including: 1) Quadrupole: A quadrupole mass analyzer uses electric fields to filter ions based on their m/z ratio, allowing for the analysis of a wide range of compounds with good sensitivity and resolution (He L. et al., 2020). 2) Time-of-Flight (TOF): TOF analyzers measure the time it takes for ions to travel a fixed distance, with lighter ions reaching the detector more quickly than heavier ions. This technique offers high resolution and is capable of measuring ions with a wide range of m/z ratios (Wang J. G. et al., 2023). 3) Orbitrap: Orbitrap analyzers trap ions in an electrostatic field and measure their frequency of oscillation to determine their m/z ratio. They provide high resolution and high mass accuracy, making them ideal for complex drug studies and metabolomics (Deslignière et al., 2023).
(3) Detection: The ions are then detected by various methods such as electron multipliers or ion traps. The detected ions are converted into a mass spectrum, which is a plot of ion intensity against m/z ratio. The resulting spectrum provides crucial information on the molecular weight, structure, and concentration of the analyte. MS’s ability to precisely measure the mass-to-charge ratio of ions enables the identification of drug molecules and their metabolites with high specificity (Anyaeche et al., 2023). Moreover, tandem MS (MS/MS) techniques, where ions are fragmented and analyzed in multiple stages, provide further structural information and allow for detailed characterization of complex molecules such as drug metabolites. Notably, UHPLC-MS/MS provides semi-quantitative data that is extremely valuable in drug research, as it allows for rapid comparative assessments of metabolite concentrations and biomarker profiling without the strict need for fully quantitative calibration. This semi-quantitative nature significantly facilitates large-scale screening studies and biomarker discovery, providing essential preliminary data for more targeted quantitative analyses (Ventura et al., 2020).

### 2.3 The development of chromatography-MS: a historical perspective

The development of chromatography-MS began in the mid-20th century, driven by the need for more advanced techniques to analyze increasingly complex drug molecules. Early work in chromatography was focused on improving separation techniques, with the invention of LC in the 1950s and GC shortly thereafter. While both techniques were valuable in separating mixtures, it was not until the integration of MS that the full potential of chromatography was realized (Arslan et al., 2022).

MS itself has a longer history, with the first mass spectrometer being developed in the early 1900s. However, it was not until the 1970s and 1980s that significant advancements were made in the coupling of chromatography with MS, particularly with the introduction of new ionization techniques like ESI and APCI. These innovations made it possible to analyze large, polar biomolecules, which were previously difficult to ionize, and enabled the application of chromatography-MS to a wide range of drug research areas (Morato and Cooks, 2023).

Over the last few decades, both chromatography and MS technologies have advanced rapidly. In particular, the development of UHPLC and the introduction of high-resolution mass spectrometers, such as Orbitrap and quadrupole time-of-flight (QTOF) instruments, have significantly improved the sensitivity, resolution, and speed of chromatography-MS analysis. Additionally, improvements in software for data acquisition, peak detection, and quantitative analysis have made it easier for researchers to handle and interpret complex datasets, further advancing the utility of chromatography-MS in drug research. Compared with earlier chromatographic methods, such as traditional HPLC, UHPLC offers greatly enhanced separation speed and peak resolution due to its smaller particle size and higher operating pressures. Likewise, early generations of mass spectrometers often suffered from limited sensitivity, mass accuracy, and fragmentation capabilities, restricting their utility for complex biological samples (Volpi et al., 2024). Modern mass spectrometers like Orbitrap and QTOF instruments have dramatically improved these aspects by providing higher resolution, mass accuracy, sensitivity, and comprehensive structural information (Nahar et al., 2024). The combination of advanced chromatography with modern MS techniques has thus overcome previous limitations, allowing for the detailed analysis of extremely complex biological matrices, detection of low-abundance metabolites, and accurate elucidation of molecular structures and pathways, significantly enhancing drug discovery and development processes.

### 2.4 The role of chromatography-MS in modern drug research

Today, chromatography-MS is an indispensable tool in drug research, offering robust and reliable analysis for a variety of applications. From drug discovery to clinical trials, chromatography-MS enables researchers to gain detailed insights into drug pharmacokinetics, metabolic pathways, and safety profiles (Gilardoni et al., 2022; Olsen et al., 2015). The technology’s ability to handle complex biological matrices, coupled with its high sensitivity and resolution, has made it an essential method for analyzing drugs, their metabolites, and their interactions within biological systems. In drug research, chromatography-MS is used to study the absorption, distribution, metabolism, and excretion (ADME) of drugs, providing invaluable data on the fate of a drug in the body and helping to predict its therapeutic efficacy and safety. In addition, chromatography-MS is a critical tool in the development of personalized medicine, allowing for the identification of biomarkers, optimizing drug dosages, and predicting patient responses to therapy.

As both chromatography and MS technologies continue to advance, the potential applications of chromatography-MS in drug research will continue to expand, offering new opportunities to accelerate drug discovery, improve safety profiles, and develop more targeted and effective therapies.

## 3 Innovative applications of chromatography-MS in drug research

### 3.1 Drug absorption and bioavailability

Understanding drug absorption is critical in drug development, as it determines the drug’s bioavailability and its therapeutic effectiveness (Chryssafidis et al., 2021). Chromatography-MS has become indispensable in studying the pharmacokinetics of drugs, including their absorption rates and profiles. By coupling liquid chromatography with MS, researchers can track the drug’s movement from the gastrointestinal tract into the bloodstream, monitor metabolites, and quantify concentrations over time (Wang P. et al., 2023).

#### 3.1.1 Innovative approaches in chromatography-MS for drug research

Recent advancements in chromatography-MS have introduced innovative methodologies that significantly enhance our understanding of drug absorption, distribution, and localization. These approaches offer valuable insights into drug behavior at both the molecular and cellular levels, ultimately contributing to more effective drug development and personalized treatment strategies.

##### 3.1.1.1 In vitro gastrointestinal models

One of the innovative applications of chromatography-MS is its integration with *in vitro* gastrointestinal (GI) models to simulate human drug absorption (Arian et al., 2022). In these models, researchers mimic the physiological conditions of the human GI tract, including variations in pH, enzyme activity, and digestive fluids, to simulate how drugs are absorbed during the digestive process (Shi et al., 2021). By coupling these models with chromatography-MS, researchers can analyze drug samples from simulated gastric and intestinal environments, quantifying both the parent drug and its metabolites with high precision (Liu et al., 2023). Recent studies have showcased the benefits of combining *in vitro* GI models with chromatography-MS for predicting human drug absorption. Research by Zhang et al. (2023) used chromatography-MS to analyze drug absorption in simulated human gastrointestinal environments, which resulted in more accurate predictions of pharmacokinetic profiles, particularly for poorly soluble compounds. This work has highlighted how improvements in simulation accuracy and sensitivity can reduce the risk of late-stage clinical failures due to absorption issues (Zhang et al., 2023). Furthermore, it aids in understanding the effects of gastrointestinal enzymes and transporters on drug bioavailability, making it an essential tool for early-stage drug development. Chromatography-MS has been used to study the absorption of poorly soluble drugs, revealing how different formulations or excipients can affect their solubility and absorption efficiency (Tran et al., 2020). This has profound implications for optimizing drug formulations to achieve desired therapeutic outcomes and improve the predictability of human clinical responses.

##### 3.1.1.2 Spatial mass spectrometry imaging

Another cutting-edge application of chromatography-MS in drug research is spatial mass spectrometry imaging (MSI), particularly with MALDI imaging mass spectrometry. This technique enables the visualization of drug distribution within biological tissues with high spatial resolution, offering insights into drug permeation and tissue localization at the cellular level (Mund et al., 2022). Unlike traditional methods that only provide bulk data, spatial MSI allows for the analysis of drug distribution across different tissue structures, revealing patterns that were previously difficult to detect (Baijnath et al., 2022).

MALDI-IMS has been particularly valuable in drug distribution studies, as it enables researchers to examine the spatial distribution of drugs and their metabolites directly in tissue sections without the need for labels or external markers (Prideaux et al., 2018). This approach has been used to study drug delivery in organs such as the brain, liver, and kidney, providing detailed information on the pharmacokinetics and pharmacodynamics of drugs in specific tissue environments (Wang et al., 2021; Sun et al., 2023; Unsihuay et al., 2021). It also aids in understanding how drugs are absorbed across barriers such as the BBB or the intestinal epithelium (Vallianatou et al., 2018). Similarly, advancements in spatial MSI have opened new doors for drug distribution studies. For example, the work of Smith et al. demonstrated the ability of MALDI-IMS to map the distribution of anticancer drugs in tumor tissues, providing critical information on drug penetration and target engagement within heterogeneous tumor microenvironments. This has the potential to revolutionize cancer therapy by enabling more precise dosing strategies and minimizing side effects (Smith et al., 2024).

The spatial resolution of MALDI-IMS allows for cellular and subcellular mapping of drug compounds, offering a unique advantage in studying the mechanisms of drug action and toxicity at the tissue level (Grüner et al., 2016). This technique has proven to be invaluable in studying drug efficacy, as it can reveal where and how drugs interact with their targets *in situ*, providing a more comprehensive picture of drug activity (Jucker et al., 2017). Furthermore, when combined with other analytical techniques, such as quantitative chromatography-MS, spatial MSI can offer a multi-dimensional approach to studying drug behavior in complex biological systems (Luo L. et al., 2023).

These innovative applications underscore the growing potential of chromatography-MS in enhancing drug development. By offering more accurate predictions of drug absorption and providing detailed, spatially resolved data on drug distribution, these technologies are poised to play a key role in the future of personalized medicine and drug efficacy studies.

### 3.2 Drug distribution and pharmacokinetics

Understanding drug distribution is a key component of pharmacokinetics and is essential for predicting the therapeutic efficacy and safety profile of a drug. Drug distribution refers to the process by which a drug moves from the bloodstream to various tissues and organs. It is influenced by factors such as blood flow, tissue permeability, and protein binding. To gain a comprehensive understanding of these processes, it is essential to accurately assess the concentration and distribution of drugs throughout the body (Peng et al., 2023).

Chromatography-MS, as a highly sensitive and precise analytical technique, has become a critical tool in studying drug distribution. By combining chromatographic separation with mass spectrometric detection, researchers can quantify drug concentrations in different tissues and organs, helping to elucidate the pharmacokinetic profile of a drug (Li et al., 2018). This includes information on how quickly the drug is absorbed, how it is distributed across tissues, how it is metabolized, and how it is ultimately excreted. The ability to analyze drug distribution in a variety of biological matrices, including plasma, tissues, and organs, allows for a better understanding of drug action and the prediction of both therapeutic and adverse effects. Furthermore, chromatography-MS provides essential information regarding the drug’s bioavailability, half-life, and clearance rate, which are critical parameters in drug development and optimization.

#### 3.2.1 Innovative approaches in drug distribution and pharmacokinetics

Recent advancements in mass spectrometry, particularly in imaging technologies and single-cell analysis, have revolutionized the way drug distribution is studied at the molecular and cellular levels. These innovative approaches provide a deeper, more precise understanding of how drugs are distributed within specific tissues, cell types, and even subcellular compartments (Nishide et al., 2023).

##### 3.2.1.1 Single-cell analysis

The emergence of single-cell analysis using MSI and advanced ionization techniques, such as nano-spray ionization (nESI), has allowed for the detection of drug distribution at the individual cell level (Chen et al., 2023; Guo et al., 2024). This method offers several advantages over traditional bulk tissue analysis, as it can capture the heterogeneity of drug distribution within different cell populations, tissues, and organs.

Recent studies have demonstrated the ability to map drug uptake and localization within single cells, providing insights into how drugs interact with specific cell types or organelles (Li et al., 2023). For instance, by applying single-cell MSI, researchers can identify how a drug may selectively accumulate in certain cell types (such as cancer cells), enabling a better understanding of the mechanisms driving drug efficacy and resistance (Meng et al., 2021). This approach is particularly valuable in oncology, where it can help assess drug distribution in tumor microenvironments, identify target cells, and optimize therapeutic strategies. Single-cell analysis also provides critical data for personalized medicine, allowing for more precise drug targeting based on individual cellular profiles. This can ultimately help tailor drug therapies to individual patients, improving treatment outcomes and minimizing side effects.

##### 3.2.1.2 Dynamic Imaging

MSI has made significant strides in enabling dynamic, real-time visualization of drug distribution within biological tissues. Advanced techniques, including desorption electrospray ionization (DESI) and matrix-assisted laser desorption/ionization (MALDI) combined with high spatial resolution, allow researchers to track the movement of drugs through tissues in real time (Wang et al., 2021). The ability to dynamically monitor drug distribution during the course of treatment provides valuable insights into the temporal aspects of drug pharmacokinetics. For example, MSI enables the visualization of how drugs permeate through tissues over time, including their ability to reach target sites, such as tumors, and penetrate barriers such as the blood-brain barrier (BBB) (Marin et al., 2021). Furthermore, it helps identify areas of suboptimal drug delivery, which may lead to poor therapeutic outcomes.

Dynamic imaging also allows researchers to observe the metabolism of drugs in tissues, tracking the formation and distribution of metabolites alongside the parent drug. This is crucial for understanding not only the pharmacokinetics of the drug but also its pharmacodynamics, helping to inform dosing strategies and identify potential toxicities at the cellular level (Tang et al., 2019). The study by Gou et al. introduced a novel laser-assisted chemical transfer (LACT) technique to enhance the detection sensitivity of central nervous system (CNS) drugs in brain tissues by minimizing ionization suppression from endogenous biomolecules. LACT successfully visualized the distribution of 16 CNS drugs in the mouse brain and enabled dynamic tracking of risperidone and its metabolite over time, offering a valuable approach for assessing drug brain penetration in pharmacological research (Guo et al., 2022). The integration of single-cell analysis and dynamic imaging with chromatography-MS holds tremendous potential for advancing drug research. These technologies enable a more detailed and accurate understanding of drug behavior in tissues, enhancing our ability to predict therapeutic efficacy, optimize dosing strategies, and minimize adverse effects. By providing spatially and temporally resolved data on drug distribution and metabolism, these approaches contribute to the development of more targeted and personalized therapies, improving patient outcomes and accelerating the drug development process (Chadha et al., 2024). Moreover, the dynamic tracking of drug distribution in real time can aid in the identification of optimal drug delivery systems and formulations, ensuring that drugs reach their intended targets efficiently and safely. As these innovative approaches continue to evolve, they will play an increasingly pivotal role in shaping the future of pharmacokinetics and drug development.

### 3.3 Drug metabolism and biomarker discovery

Drug metabolism plays a pivotal role in determining the efficacy, safety, and toxicity of pharmaceutical compounds (Shao et al., 2021). Metabolic processes, including the transformation of drugs into active or inactive metabolites, are often mediated by enzymes such as cytochrome P450 (CYP450) (Zhao et al., 2021). Understanding these metabolic pathways is essential for predicting drug responses, optimizing dosing regimens, and identifying potential toxicities (Awad et al., 2024). Chromatography-MS has emerged as a critical tool in studying drug metabolism, as it allows for the precise identification and quantification of metabolites in complex biological samples, such as plasma, urine, or liver tissue (Arnold et al., 2024).

In drug metabolism studies, chromatography-MS provides detailed insights into the fate of drugs in the body. By combining the separation power of chromatography with the molecular specificity of MS, this technique enables the identification of phase I (oxidation, reduction, hydrolysis) and phase II (conjugation) metabolites, which are crucial for assessing a drug’s safety and therapeutic efficacy (Niu et al., 2024). Additionally, chromatography-MS facilitates the study of drug interactions with enzymes, helping to predict how drugs are metabolized and whether they may interfere with other medications (Dong and Zhuang, 2024). Moreover, chromatography-MS plays a significant role in biomarker discovery, as it enables the identification of biomarkers associated with drug efficacy, toxicity, and disease states (Xu et al., 2023). By providing a comprehensive analysis of metabolites, researchers can uncover biomarkers that predict patient responses to drugs, identify adverse reactions, and develop personalized medicine strategies.

#### 3.3.1 Innovative approaches in drug metabolism and biomarker discovery

##### 3.3.1.1 Metabolomics

Metabolomics is the large-scale study of metabolites, small molecules involved in cellular processes, and their dynamic changes in response to drug treatments. Chromatography-MS has become the cornerstone of metabolomics, enabling the identification and quantification of thousands of metabolites in biological samples with high sensitivity and accuracy (Luo J. et al., 2023). Metabolomics applied to drug metabolism helps identify not only the drug metabolites but also other endogenous metabolites that may be influenced by drug exposure. This approach provides a comprehensive profile of metabolic changes, allowing researchers to better understand how a drug affects cellular functions, energy metabolism, and organ systems (Jurich et al., 2024). By identifying changes in the metabolite profile, metabolomics can pinpoint biomarkers of drug efficacy or toxicity, offering valuable information for early-stage drug development and personalized treatment strategies (Jaber et al., 2024). For example, in oncology, metabolomics has been used to identify biomarkers for drug response in cancer treatment (Sun et al., 2024). Chromatography-MS has identified metabolites that correlate with tumor growth or drug resistance, providing crucial information for selecting the right drug for a specific patient. Similarly, in cardiovascular research, metabolomic analyses can uncover biomarkers associated with drug-induced toxicity, helping to avoid adverse drug reactions that could compromise patient safety.

##### 3.3.1.2 CYP450 assays

CYP450 enzymes are central to the metabolism of many drugs, and their activity is a key determinant of drug pharmacokinetics. Using chromatography-MS, researchers can study the activity of these enzymes and their role in drug metabolism. MS allows for the identification of CYP450-mediated metabolites and provides valuable data on enzyme activity, enabling researchers to predict how different drugs may interact with CYP450 enzymes.

In drug discovery, understanding CYP450-mediated metabolism is critical for assessing the potential for drug-drug interactions. For example, drugs that inhibit or induce specific CYP450 enzymes can alter the metabolism of co-administered drugs, leading to altered drug efficacy or toxicity. Chromatography-MS is frequently used to monitor CYP450 activity *in vitro*, providing insight into how drugs are metabolized and identifying potential DDIs early in the development process (Anderson et al., 2021). This information is vital for drug safety evaluations and the design of drug regimens that minimize the risk of adverse interactions. Additionally, MS-based assays for CYP450 activity are used in clinical studies to assess individual variability in drug metabolism (Grangeon et al., 2021). Genetic differences in CYP450 enzymes can lead to variations in drug response, and chromatography-MS can be used to identify these variations, paving the way for more personalized and effective drug therapies.

#### 3.3.2 Impact on drug development and personalized medicine

The integration of metabolomics and CYP450 assays with chromatography-MS has profound implications for drug development. These innovative approaches help identify key metabolites that influence drug action, predict potential adverse effects, and uncover biomarkers of drug efficacy and toxicity. By providing insights into the metabolic fate of drugs, chromatography-MS allows for the optimization of drug candidates, ensuring that they are both effective and safe.

Moreover, these technologies are central to the advancement of personalized medicine, as they provide the tools necessary to tailor drug therapies based on individual metabolic profiles. For example, metabolomics can be used to identify biomarkers that predict how a patient will respond to a particular drug, while CYP450 assays help guide the selection of drugs that are less likely to cause harmful drug-drug interactions.

In the future, the combination of metabolomics, CYP450 assays, and chromatography-MS is expected to play a critical role in streamlining the drug discovery process. These techniques will continue to enhance our understanding of drug metabolism, improve drug safety, and facilitate the development of more targeted and personalized treatments for patients.

### 3.4 Drug toxicity and side effect evaluation

Drug toxicity is a critical concern in drug development, as adverse effects can lead to the termination of promising drug candidates during clinical trials (Attwa et al., 2020). Toxicity can manifest in various forms, such as organ damage, metabolic disturbances, and immunological responses, often caused by the accumulation of toxic metabolites. Therefore, understanding the mechanisms underlying drug toxicity and identifying harmful metabolites is essential for ensuring drug safety and efficacy (Hanna et al., 2020). Chromatography-MS plays a pivotal role in toxicity assessment by enabling the identification and quantification of toxic metabolites, as well as providing insights into the effects of drugs on biological systems at the molecular level.

Chromatography-MS offers several advantages for toxicity studies, including high sensitivity, resolution, and the ability to analyze complex biological matrices, such as plasma, liver tissue, and urine (Guidolin et al., 2023). These capabilities make it an indispensable tool in the early stages of drug development, where it can be used to predict and evaluate potential toxic effects before clinical trials. By identifying and profiling toxic metabolites and proteins involved in adverse reactions, chromatography-MS helps researchers better understand the mechanisms of drug-induced toxicity and provides a foundation for safer drug design.

#### 3.4.1 Innovative approaches in drug toxicity and side effect evaluation

##### 3.4.1.1 Toxicoproteomics

The integration of proteomics with chromatography-MS, often referred to as toxicoproteomics, is an emerging approach to understanding drug toxicity at the protein level (Madeira and Costa, 2021). Toxicoproteomics combines the identification of protein expression changes, post-translational modifications, and interactions with the analysis of metabolites, providing a comprehensive view of how drugs affect cellular and tissue systems (Al Sultan et al., 2024). By analyzing protein expression profiles in response to drug exposure, researchers can identify proteins that are involved in the toxic response, including those that may act as biomarkers for toxicity. For example, drugs that cause liver damage may induce changes in proteins related to oxidative stress, inflammation, or cellular apoptosis (Nury et al., 2023). Using chromatography-MS, these changes can be detected at early stages, allowing for the identification of potential biomarkers of liver toxicity, kidney damage, or cardiovascular toxicity.

Toxicoproteomics not only helps identify toxicity mechanisms but also provides valuable insights into the pathways and cellular processes that are disrupted by drugs. This can lead to a better understanding of the broader effects of drugs on cellular function and the identification of new therapeutic targets for mitigating adverse drug reactions (Suman et al., 2016).

##### 3.4.1.2 Biomarker discovery

The identification of biomarkers that can predict or indicate drug toxicity is a key component of drug safety evaluations. Chromatography-MS plays a central role in discovering these biomarkers by providing a detailed analysis of the metabolome and proteome in response to drug exposure (Upadhyay et al., 2023; Chen et al., 2016). Metabolomic and proteomic profiling can reveal subtle changes in cellular or metabolic pathways that are associated with toxicity, even before clinical symptoms manifest. For instance, the detection of certain metabolites or proteins in blood or urine samples can indicate early signs of organ damage or systemic toxicity, enabling more effective monitoring and intervention (Qian et al., 2018). In cancer therapy, for example, certain biomarkers can be tracked to monitor the onset of chemotherapy-induced nephrotoxicity or hepatotoxicity. This allows clinicians to adjust dosages or switch treatments early, improving patient safety and reducing the risk of serious adverse events (Qi et al., 2019).

Additionally, biomarkers can be used to identify individuals who may be more susceptible to drug toxicity due to genetic variations or pre-existing conditions. Through the use of chromatography-MS, personalized approaches to drug toxicity assessment can be developed, ensuring that drugs are tailored to individual patient profiles, minimizing risks, and maximizing therapeutic efficacy.

#### 3.4.2 Impact on drug safety and development

The integration of toxicoproteomics and biomarker discovery with chromatography-MS enhances the ability to assess drug toxicity and predict adverse reactions, reducing the risks associated with new drug candidates. These advanced analytical methods allow researchers to identify toxicity risks early in the drug development process, optimizing safety profiles before drugs enter clinical trials.

Furthermore, these techniques offer the potential to develop safer drugs by identifying biomarkers of toxicity that can be used in preclinical and clinical testing. This allows for more informed decisions about the progression of drug candidates and the design of safer therapies. By revealing the underlying molecular mechanisms of drug-induced toxicity, chromatography-MS also aids in the development of strategies to mitigate side effects, improving patient outcomes and enhancing the overall drug development process.

Ultimately, the continued use and refinement of chromatography-MS for toxicity studies will play an important role in accelerating drug development while ensuring that only the safest, most effective drugs reach the market. These innovations in drug safety evaluation will contribute to more personalized and targeted therapies, improving the risk-benefit ratio of new treatments and advancing the field of precision medicine.

## 4 Chromatography-MS in personalized medicine

The advancement of personalized medicine has revolutionized how healthcare is delivered, shifting the focus from a “one-size-fits-all” approach to treatments that are tailored to the individual characteristics of each patient (Gastmeier, 2020). Personalized medicine aims to optimize treatment strategies by considering factors such as genetic makeup, lifestyle, and environmental influences (Cui et al., 2020). Chromatography-MS is playing an increasingly vital role in this paradigm shift, as it enables the analysis of patient-specific biological profiles that can guide more precise and effective drug therapies (Kowalczyk et al., 2020; Zhang et al., 2018). By integrating data from genomics, proteomics, and metabolomics, chromatography-MS provides comprehensive insights into how patients respond to drugs at the molecular level.

One of the key challenges in personalized medicine is understanding the variability in drug response among individuals, which can be attributed to genetic differences, variations in metabolism, or differences in disease pathology (Jove et al., 2019). Chromatography-MS addresses this challenge by offering a robust platform to identify patient-specific biomarkers, metabolites, and proteins, enabling clinicians to tailor drug regimens that optimize therapeutic outcomes and minimize adverse effects. Through the integration of MS with other omics technologies, researchers and clinicians can develop a detailed understanding of an individual’s metabolic fingerprint, leading to the development of precision pharmacotherapies that are more effective and safer. This approach has profound implications for fields such as oncology, cardiology, and infectious diseases, where patient-specific factors can significantly impact treatment efficacy.

### 4.1 Precision pharmacotherapy

Precision pharmacotherapy refers to the customization of drug therapies based on a patient’s unique biological and metabolic profile. Chromatography-MS plays a pivotal role in this process by providing detailed metabolic and proteomic data that can be used to select the most appropriate drugs and dosages for individual patients. By analyzing biological samples (e.g., blood, urine, tissue), chromatography-MS allows for the identification of metabolic pathways, enzymes, and biomarkers that influence drug metabolism and efficacy (Meng et al., 2021). For example, in cancer treatment, patients with the same cancer type may respond differently to chemotherapy based on their genetic mutations, metabolic profiles, or drug interactions. Using chromatography-MS to analyze a patient’s tumor metabolites, researchers can identify biomarkers that predict whether a specific chemotherapy regimen will be effective or if alternative therapies may be necessary. This approach not only improves treatment outcomes but also minimizes the risk of adverse effects, as drugs can be carefully selected and dosed based on individual metabolic capabilities (Plana et al., 2022). Furthermore, in the treatment of chronic diseases such as diabetes or cardiovascular disorders, chromatography-MS can be used to monitor metabolic responses to treatment, allowing for adjustments to therapy based on real-time metabolic data. This enables clinicians to better manage disease progression, optimize drug efficacy, and reduce the likelihood of side effects.

### 4.2 Pharmacogenomics

Pharmacogenomics is the study of how genetic variations affect an individual’s response to drugs (Roden et al., 2019). The integration of chromatography-MS with pharmacogenomic data has become an essential tool for personalizing drug treatment strategies. By combining MS-based techniques with genetic analysis, it is possible to measure drug-metabolizing enzyme activity, transporter function, and genetic polymorphisms that influence drug metabolism (Relling and Evans, 2015). These insights allow clinicians to customize dosing regimens based on a patient’s genetic makeup, improving therapeutic efficacy and reducing the risk of adverse drug reactions. For instance, some patients may have genetic variations in CYP450 enzymes, which are involved in drug metabolism (Hayes and Rae, 2020). These variations can lead to faster or slower metabolism of certain drugs, affecting drug efficacy and the risk of toxicity. Using chromatography-MS, clinicians can measure the specific activity of these enzymes in a patient’s system and adjust the drug dosage accordingly (Stingl, 2018). Similarly, MS can be used to assess the activity of drug transporters, proteins that regulate the absorption and distribution of drugs within the body. By measuring transporter activity, personalized dosing strategies can be developed to ensure optimal drug delivery to the target tissue (Ahmed et al., 2016). In oncology, pharmacogenomic approaches combined with chromatography-MS are particularly promising. Genetic variations in both the patient and the tumor itself can influence how a patient responds to cancer treatments. For example, certain mutations in the epidermal growth factor receptor (EGFR) gene can affect the efficacy of targeted therapies like erlotinib (Kolesar et al., 2022; Chen et al., 2020). By combining MS-based analysis of tumor metabolism with pharmacogenomic information, clinicians can more accurately predict which patients will respond to specific treatments, enabling more precise and personalized cancer care (Grossman et al., 2017).

The integration of chromatography-MS with precision pharmacotherapy and pharmacogenomics not only improves the safety and efficacy of drug therapies but also helps reduce healthcare costs by avoiding ineffective treatments and minimizing adverse drug reactions (Lin et al., 2024). Personalized medicine powered by chromatography-MS ensures that patients receive the right drug at the right dose, based on their unique genetic and metabolic characteristics. As the technology advances, chromatography-MS will play an even more significant role in the development of precision drugs, enabling the creation of therapies that are specifically tailored to the genetic and metabolic profiles of patients. This approach holds the potential to revolutionize the treatment of complex diseases, offering more targeted, effective, and safer options for patients worldwide.

In conclusion, chromatography-MS is a powerful tool for advancing personalized medicine, and its application in precision pharmacotherapy and pharmacogenomics is transforming the way drugs are developed, prescribed, and monitored. By providing comprehensive insights into the molecular factors that influence drug response, this technology is paving the way for a future where treatments are optimized for each individual, leading to better clinical outcomes and improved patient care.

## 5 Future perspectives and challenges

Despite the tremendous progress made in the development and application of chromatography-MS in drug research, several challenges remain that hinder its broader adoption and optimal utilization. These challenges, while not insurmountable, require continuous innovation and improvement to enhance the overall impact of chromatography-MS in drug discovery, development, and personalized medicine. Key limitations include the need for improved sensitivity, higher resolution in complex biological matrices, and more efficient data analysis techniques.

### 5.1 Key challenges in chromatography-MS

#### 5.1.1 Sensitivity and quantification in complex biological matrices

One of the most significant challenges in drug research using chromatography-MS is the ability to detect trace levels of drug molecules and metabolites in complex biological matrices such as plasma, tissues, and urine. In drug metabolism and toxicity studies, low-abundance compounds, such as metabolites or toxic by-products, must be accurately quantified to assess the pharmacokinetics and safety profiles of drugs. Enhancing the sensitivity of mass spectrometers and improving the separation capabilities of chromatography will be crucial for detecting these compounds at trace levels and increasing the reliability of results in clinical studies.

#### 5.1.2 Resolution in complex biological samples

Biological samples, particularly plasma and tissue extracts, often contain hundreds or thousands of compounds that can interfere with the analysis of the target molecules. Despite significant advancements in the resolution of chromatography-MS, the ability to resolve highly complex mixtures-such as those encountered in pharmacokinetic or toxicological studies-remains a challenge. Improved chromatographic separations, coupled with more sophisticated MS techniques, are needed to provide higher resolution and greater sensitivity for resolving these complex biological matrices.

#### 5.1.3 Data analysis and interpretation

The volume of data generated by chromatography-MS, especially in high-throughput studies, is immense and can be difficult to interpret without sophisticated computational tools. Current data analysis methods may not be efficient enough to process the vast amounts of data generated during drug research, which can delay the identification of relevant biomarkers or drug-metabolizing pathways. More robust, user-friendly software and algorithms are needed to handle these complex datasets and make them accessible to researchers across different fields of drug research.

#### 5.1.4 Standardization and reproducibility

An additional critical challenge facing chromatography-MS is the lack of standardized protocols and guidelines across laboratories and research institutions. Due to diverse methodologies, varying instrumentation, and different sample preparation techniques, results obtained in chromatography-MS studies often exhibit significant variability, hindering comparability and reproducibility. This lack of standardization poses substantial obstacles, particularly in regulatory submissions, clinical research, and large-scale multi-center studies. To address these challenges, efforts such as those by the Metabolomics Standards Initiative have highlighted the importance of standardized reporting guidelines and quality control measures for chromatography-MS workflows (Sumner et al., 2007). Encouraging the adoption of uniform analytical protocols, robust reference materials, and transparent data reporting practices across the research community will significantly enhance reproducibility, data comparability, and the overall reliability of chromatography-MS applications in drug research.

### 5.2 Future directions in chromatography-MS

Despite these challenges, several exciting directions for the future of chromatography-MS are emerging, which hold promise for overcoming current limitations and further enhancing its capabilities in drug research.

#### 5.2.1 High-resolution imaging and multimodal techniques

One of the most promising directions for future research is the integration of chromatography-MS with high-resolution imaging techniques. Combining MS with magnetic resonance imaging, fluorescence microscopy, and optical imaging will allow researchers to visualize drug distribution, metabolism, and toxicity in living tissues at the cellular and subcellular levels. MSI, for example, is already providing high spatial resolution images of drug distribution in tissue slices, but when combined with other imaging modalities, it can give more detailed insights into how drugs interact with tissues in real time. These multimodal techniques will provide a more comprehensive understanding of drug mechanisms of action and enable a more precise evaluation of drug efficacy and safety at the cellular level.

The integration of spatial transcriptomics or proteomics with MSI techniques, for instance, could help to identify biomarkers of drug action in specific tissue regions, thus advancing our understanding of the molecular drivers of drug response. Additionally, coupling chromatography-MS with multimodal imaging technologies will enhance the study of complex biological phenomena such as drug-target interactions, tissue-specific drug metabolism, and drug-induced cellular damage, which are often difficult to capture with a single analytical approach.

#### 5.2.2 Artificial intelligence and machine learning

As chromatography-MS continues to produce increasingly large and complex datasets, the integration of artificial intelligence (AI) and machine learning (ML) into data analysis holds great promise for improving the speed, accuracy, and reproducibility of results. AI and ML algorithms can analyze vast amounts of data to identify patterns, correlations, and anomalies that may not be apparent through traditional analytical techniques. For instance, ML models can be trained to predict drug efficacy, metabolism, and toxicity based on historical data and patient-specific profiles, significantly reducing the time needed to identify promising drug candidates. Several existing AI/ML-based approaches illustrate this potential clearly. For example, convolutional neural networks (CNNs) and random forest algorithms have been successfully implemented for peak analysis, alignment, and compound identification in GC-MS datasets, significantly improving data processing speed and accuracy (Domingo-Almenara et al., 2018). In addition, deep learning approaches such as DeepLC and MetaboAnalyst have demonstrated great promise in untargeted LC-MS metabolomics by facilitating accurate metabolite identification, retention time prediction, and biomarker discovery (Bouwmeester et al., 2021; Pang et al., 2022). These examples underscore how the application of AI and ML is poised to revolutionize chromatography-MS by enabling more efficient workflows and deeper insights into complex biological systems.

AI-powered tools could also enable the real-time analysis of chromatography-MS data during drug discovery, allowing researchers to make faster decisions regarding drug development. By automating data interpretation and providing insights based on predictive modeling, AI and ML can also reduce human errors and subjectivity in data analysis, increasing the consistency and reliability of results. Furthermore, ML algorithms can optimize chromatographic separation and mass spectrometry methods, helping researchers select the most suitable experimental parameters for specific drug studies. This could lead to more efficient workflows, reducing experimental time and costs, while also improving the reproducibility of results across different laboratories and research settings.

#### 5.2.3 Advances in sample preparation and automation

One additional area of development for chromatography-MS lies in the optimization of sample preparation and automation. As biological samples often require extensive preparation before being analyzed, the introduction of automated systems for sample collection, extraction, and cleanup could reduce sample loss, minimize human error, and streamline high-throughput screening processes. Automating these steps, particularly in large-scale studies, will help researchers manage vast numbers of samples and accelerate the pace of drug discovery, allowing for the analysis of more diverse drug candidates under a variety of experimental conditions.

#### 5.2.4 Real-time monitoring and personalized medicine

Real-time monitoring of drug levels, metabolism, and toxicity using chromatography-MS will become increasingly important as we move toward more personalized drug therapies. As patient-specific profiles become integral to drug treatment plans, the ability to monitor and adjust drug regimens in real-time will be essential. Future chromatography-MS advancements will allow clinicians to better track how drugs are distributed and metabolized in individual patients, enabling more precise adjustments to doses and treatment strategies based on real-time biological data.

## 6 Conclusion

The future of chromatography-MS in drug research is bright, with significant opportunities for innovation and improvement. While current challenges exist, advancements in multimodal imaging, AI, and sample automation are poised to enhance the capabilities of chromatography-MS, making it an even more powerful tool in drug discovery and personalized medicine. These advancements will not only increase the efficiency and accuracy of drug development but will also contribute to the development of safer, more effective treatments tailored to individual patients. As these technologies continue to evolve, chromatography-MS will remain at the forefront of transformative changes in the way drugs are discovered, developed, and delivered to patients.
